# Public health economic evaluation of different European Union–level policy options aimed at reducing population dietary *trans* fat intake^1^,^2^

**DOI:** 10.3945/ajcn.116.136911

**Published:** 2016-09-28

**Authors:** Carlos Martin-Saborido, Theodora Mouratidou, Anastasia Livaniou, Sandra Caldeira, Jan Wollgast

**Affiliations:** European Commission, Joint Research Centre (JRC), Ispra, Italy

**Keywords:** European Union, cost-effectiveness, public health, public policy, *trans* fatty acids

## Abstract

**Background:** The adverse relation between dietary *trans* fatty acid (TFA) intake and coronary artery disease risk is well established. Many countries in the European Union (EU) and worldwide have implemented different policies to reduce the TFA intake of their populations.

**Objective:** The aim of this study was to assess the added value of EU-level action by estimating the cost-effectiveness of 3 possible EU-level policy measures to reduce population dietary TFA intake. This was calculated against a reference situation of not implementing any EU-level policy (i.e., by assuming only national or self-regulatory measures).

**Design:** We developed a mathematical model to compare different policy options at the EU level: *1*) to do nothing beyond the current state (reference situation), *2*) to impose mandatory TFA labeling of prepackaged foods, *3*) to seek voluntary agreements toward further reducing industrially produced TFA (iTFA) content in foods, and *4*) to impose a legislative limit for iTFA content in foods.

**Results:** The model indicated that to impose an EU-level legal limit or to make voluntary agreements may, over the course of a lifetime (85 y), avoid the loss of 3.73 and 2.19 million disability-adjusted life-years (DALYs), respectively, and save >51 and 23 billion euros when compared with the reference situation. Implementing mandatory TFA labeling can also avoid the loss of 0.98 million DALYs, but this option incurs more costs than it saves compared with the reference option.

**Conclusions:** The model indicates that there is added value of an EU-level action, either via a legal limit or through voluntary agreements, with the legal limit option producing the highest additional health benefits. Introducing mandatory TFA labeling for the EU common market may provide some additional health benefits; however, this would likely not be a cost-effective strategy.

## INTRODUCTION

*trans* Fatty acids (TFAs)^4^ are a type of unsaturated fatty acid that have ≥1 unsaturated, nonconjugated double bond in the *trans* configuration. TFA intake can be of industrial (mainly partially hydrogenated oils) or natural (ruminant food sources) origin (1). The detrimental effects of dietary intake of industrially produced TFAs (iTFAs) on heart health were first reported in the 1990s (2) and are now well established (3–5). Other health effects have been attributed to iTFA intake, such as on insulin sensitivity, obesity, diabetes, cancer, or early growth and development (3, 6). Most official guidelines recommend limiting daily TFA intake as much as possible within an adequate diet or to intakes of <1% or 2% of total energy (E%) (7). Many countries worldwide have policies to reduce population TFA intake (8); these are accompanied by significant reductions in food TFA content, with the largest reductions being observed in situations in which legal limits on TFAs are in place (9).

In the European Union (EU), dietary TFA intake has been decreasing since the 1980–1990s, from as high as 4.3 E% in elderly Dutch men in 1985 (9) to average population intakes <1 E% in the 2000s (1, 10, 11). These estimates include both iTFAs and TFAs from ruminant sources, with the latter contributing between 0.3 and 0.8 E% depending on dietary habits (11). Although less is known about dietary TFA intakes in Eastern Europe, data on TFA content of selected foods sampled between 2005 and 2014 suggest somewhat higher amounts than in most other parts of Europe (12–14). Recent data also suggest that the reduction in iTFAs in foods continued in some, but not all, European countries from 2006 to 2013 (13) and 2012 to 2014 (12).

Several health economic models suggest that reducing population iTFA intakes provides health benefits [i.e., reductions in cardiovascular disease or coronary artery disease (CAD)–related events and deaths as well as cost savings] (15–18). Restrepo and Rieger (19) estimated that the 2004 legal limit on iTFAs in Denmark has prevented ∼14.2 deaths · 100,000 persons^−1^ · y^−1^. Another study suggests that introducing a legal limit on iTFAs in England would prevent ∼7200 deaths from CAD (or 2.6% of all predicted CAD deaths) between 2015 and 2020, providing the greatest health benefits and reduction in the inequality gap when compared with improved TFA labeling or TFA removal from restaurants and fast foods (20). Because the EU and its member states are currently evaluating the impact of possible measures at the EU level (21, 22), this study presents an economic evaluation to compare the cost-effectiveness of 3 different policy options against the option of taking no action at the EU level (reference situation).

## METHODS

### Model development

**FIGURE 1:**
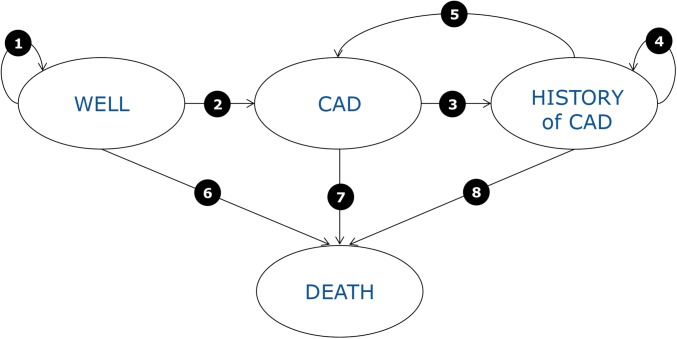
Schematic representation of the Markov model used to simulate how people move in yearly cycles through 4 health states in each of the policy options. The 4 health states are as follows: “Well” (the state for each individual with no history of CAD; a person can remain here until death or move to “CAD”); “CAD” (individuals who have CAD move to this state for a maximum of 1 y; from this state, individuals can move either to “History of CAD” or “Death” but not back to the “Well” state); “History of CAD” (post–acute CAD health state; survivors from a “CAD” state move to this state until death or until they suffer a new CAD event, in which case they move to the “CAD” state); and “Death” [this is an absorbing state (once a person enters this state, they cannot leave it); any individual can move to “Death” at any time]. The meaning of each transition probability between health states is as follows: *1*) probability of keeping well (in this context, staying alive and not having a CAD event), *2*) probability of experiencing a CAD event for persons without a previous CAD event, *3*) probability of surviving a CAD event, *4*) probability of staying alive in the post–acute CAD state, *5*) probability of experiencing a new CAD event when in the post–acute CAD state, *6*) probability of death from any cause (except from CAD) for persons without a previous CAD event, *7*) probability of death from a CAD event, and *8*) probability of death from any cause except for CAD for individuals with a history of CAD. CAD, coronary artery disease.

We developed a computer-simulated, Markov, state-transition model with the use of Excel (Microsoft Office 2010). This type of model is appropriate because Markov models are suitable for changing systems (i.e., where there is movement or transitions between different states). In this case, the different states are the conditions in which an individual can be, such as “well,” with “CAD” or “history of CAD,” or “dead” (see **Figure 1**). In addition, because the available data are population-based, discrete simulation models cannot be used and a cohort model such as Markov should be chosen instead. The International Society for Pharmacoeconomics and Outcome Research-Society for Clinical Decision Making (ISPOR-SMDM) Modeling Good Research Practices Task Force recommends Markov models for this kind of analysis (23, 24).

The TFA intake, defined as E%, as a starting point for the model (“today”) was calculated as described in **Supplemental Tables 1–3**). The model was applied to the EU population and accounts for all costs and effects applicable or resulting from the following policy options over the course of a lifetime (85 y) (25):

*1*) Reference situation (no action at the EU level): The reference situation is described by the highest cumulative TFA intake (i.e., the highest population TFA intake when summing up yearly population TFA intakes over the modeled time horizon of 85 y) in all of the 4 options, and therefore it also entails the highest risk of CAD. Nevertheless, even for this case of “no action at EU level,” in the model we assume a continued decrease in TFA consumption that leads to a removal of iTFAs from the food supply over 10 y due to continuous innovation in the industry and efforts at the national or regional levels. In terms of costs, there are no added public costs from implementing this policy option; all costs result from CAD-associated morbidity and loss of productivity.
*2*) Voluntary agreements: With this option policy makers actively seek agreements at the EU level, such as with the food industry and retailers to introduce measures that reduce TFA amounts in foods and/or between EU member states, to agree on a common framework toward reducing TFAs in foods and diets similarly to the EU salt reduction framework (26). In this case, public costs are CAD-associated and are also related to food inspection programs to monitor and evaluate the agreements. We assume a faster reduction in TFA consumption than in option 1, leading to a quicker removal of iTFAs from the food supply due to the additional private-public commitments. For this strategy in the model we assume the total removal of iTFAs from the food supply after 5 y, half the time needed in the absence of EU-level action (reference situation), albeit acknowledging that the rare use of iTFA-containing raw materials by some producers and imports of iTFA-containing foods from countries in which the iTFA issue has not been addressed cannot be excluded.
*3*) Mandatory TFA labeling: With this strategy the existing rules for the nutrition declaration on foods as governed by EU regulation 1169/2011 would be changed to require the disclosure of the TFA contents in all prepackaged foods. This provides an incentive for food reformulation toward reducing or replacing iTFAs, but only for prepackaged foods. Because this option requires legislative action, in addition to CAD-associated public costs, other non–CAD-related public costs are also considered. These are linked to the implementation of the legislation (mass media costs), worksite interventions, consumer education, and nutrition counseling as well as food inspection (9). The reduction in population TFA intake is faster than in the reference situation but slightly slower than in option 2 (voluntary agreements), because in this case there are only incentives toward reducing TFA content in prepackaged foods. The assumption in the model is that iTFA removal is faster in prepackaged foods than in options 1 (reference) and 2, but not in non-prepackaged foods, in which iTFA removal proceeds at the same speed as in the reference option 1. The model assumes population TFA intake reductions for the first 2 y until TFA content labeling is available for all prepackaged foods, as in the reference situation (option 1), then a faster reduction in iTFA intake from prepackaged foods, which, based on the available information (see Supplemental Table 2), is assumed to contribute to 50% of population TFA intake at the start and decrease to 0% in 3 y. The model assumes that reductions in iTFAs from non-prepackaged foods continue at the same speed as in the reference situation albeit acknowledging that, in reality, some spillover effects in the efforts to remove iTFAs from prepackaged foods might also be expected for non-prepackaged foods.
*4*) Legal limit of iTFA content in foods: This option sees a restriction in the use of iTFAs in the food supply through a legislative limit, such as that already introduced by some EU member states (Denmark, Austria, Hungary, and Latvia). This measure results in a fast removal of iTFAs in all of the EU food supply and represents therefore the lowest cumulative TFA consumption of all 4 options. The model assumes the total removal of iTFAs in 2 y. This strategy implies, in addition to CAD-associated public costs, other costs that are not associated with CAD such as public costs for food inspection programs.

The model simulates how people are moving in yearly cycles through 4 health states, as shown in Figure 1. Costs (of policy implementation and CAD-related) and effects [CAD incidence and disability-adjusted life-years (DALYs)] are accounted for as the population circulates through the model. These are calculated for each policy option and then compared with one another. An annual discount rate of 3.5% is applied to both costs and effects following best-practice guidelines (25). The economic evaluation presented here is broader than a simple evaluation focused on health system costs because it also includes a societal perspective: the costs included are not only health care–related costs but also indirect costs stemming from informal care and loss of productivity due to mortality and morbidity as well as policy implementation–related public costs.

### Costs

All of the costs considered to account for the burden of TFAs have been adjusted for inflation to 2011 prices (in €); currency exchanges, when applicable, were calculated on the basis of 1 January 2011 exchange rates to the euro. The model considers 3 types of costs (see also **Table 1**):

- Health care costs: These costs stem from the use of health resources (i.e., primary care costs, outpatient costs, emergency costs, and medication used during the hospitalization). The costs are based on the European Cardiovascular Disease Statistics 2012 (28).
- Non–health care costs: This group of costs includes all non–health care costs related to the disease, namely loss of productivity and informal care. The costs are based on the European Cardiovascular Disease Statistics 2012 (28).
- Costs of policy-associated measures: Each policy option (apart from option 1 “reference situation”) incurs costs related to the execution of measures needed for their successful implementation.

**TABLE 1:** Model variables and their source, deterministic value, and distribution type^1^

Variable description	Source (reference)	Deterministic value	Distribution^2^
Probability of death from acute CAD	Data from HFA-DB (27)	Life tables	Log-normal
Probability of death from any cause	Data from HFA-DB (27)	Life tables	Log-normal
Probability of CAD	Hospital discharges by IHD; data from HFA-DB (27)	Morbidity table	Log-normal
Reduction in RR of CAD in legal limit strategy	Mozzafarian et al. (4), O'Flaherty et al. (18)	Age and sex dependent	Log-normal
Reduction in RR of CAD in voluntary agreements strategy	Mozzafarian et al. (4), O'Flaherty et al. (18)	Age and sex dependent	Log-normal
Reduction in RR of CAD in mandatory labeling strategy	Mozzafarian et al. (4), O'Flaherty et al. (18)	Age and sex dependent	Log-normal
RR of second and subsequent CAD events after the first event	Assumption	1.5	Log-normal
RR probability of death from second CAD event compared with death from the first CAD event	Assumption	1.5	Log-normal
Production losses due to mortality	Nichols et al. (28)	€5101.94	γ
Production losses due to morbidity	Nichols et al. (28)	€2158.88	γ
Informal care	Nichols et al. (28)	€6440.19	γ
Primary care	Nichols et al. (28)	€617.34	γ
Outpatient care	Nichols et al. (28)	€854.56	γ
Accident and emergency	Nichols et al. (28)	€213.78	γ
In-patient care	Nichols et al. (28)	€3557.46	γ
Medication	Nichols et al. (28)	€1605.36	γ
School-based intervention	Cecchini et al. (29), Sassi et al. (30)	€1.15	γ
Worksite intervention	Cecchini et al. (29), Sassi et al. (30)	€4.48	γ
Mass media campaigns	Cecchini et al. (29), Sassi et al. (30)	€1.90	γ
Physician counseling	Cecchini et al. (29), Sassi et al. (30)	€8.28	γ
Program of food inspection	Cecchini et al. (29), Sassi et al. (30)	€0.86	γ

1 CAD, coronary artery disease; HFA-DB, Health for All Database; IHD, ischemic heart disease; TFA, *trans* fatty acid.

2 Distributions were chosen following the recommendations in reference 31.

The costs associated with each of the measures are described in the Organization for Economic Cooperation and Development report entitled “Improving lifestyles, tackling obesity: the health and economic impact of prevention strategies” (30). Costs per person per year have been adapted from this report and applied to the model to best estimate the real costs to governments (costs were adjusted by using the purchasing power parity methods to adjust for cost of living between countries). 

The following types of costs were considered (see also Table 1). The first 4 relate to the provision of information and counseling on TFAs, TFA-related health issues, and interpretation of food labels (if applicable).

*1*) School-based interventions costs: These include training teachers and food service staff and additional curricular activities, but exclude changes in food provision services.
*2*) Worksite interventions costs: These include costs from activities subsidized by the public sector and held in worksites by employers.
*3*) Mass media campaigns costs: These include broadcasting advertisements on national and local radio and television channels and for designing, producing, and distributing flyers and leaflets.
*4*) Physician counseling costs: These costs include counseling provided by physicians to targeted individuals.
*5*) Program of food inspection costs: These include the administration, planning, enforcement, and resources needed to manage food inspection.

### Effects

The model calculates, for each option, CAD events and mortality in yearly cycles over a period of 85 y. It is based on current estimates of iTFA intake (detailed in reference 32 and as shown in Supplemental Tables 1–3) and the assumed reductions in TFA intake over the years as described above. In addition, the RRs for CAD associated with the different TFA intakes are based on the calculations in Mozaffarian et al. (4) in which the “pooled multivariable-adjusted RR for 2%E of TFA, as an isocaloric replacement for carbohydrate, was 1.23 (95% CI = 1.11–1.37).” This is then applied to the different iTFA intakes to calculate the probability of a CAD event (see probability 2 in Figure 1). For the starting point of the model (“today”) the risk of CAD is calculated on the basis of hospital discharges (see explanation below) and already includes the risks from current iTFA intakes, which are specific according to country, age, and sex (Supplemental Tables 1–3). The reduction in CAD risk linked to iTFA reductions in the following years from “today” is then calculated by using the RR above.

Subsequently, the resulting DALYs are then calculated on the basis of the modeled number of CAD events and deaths. DALYs reflect, in a single quantitative figure, years of life lost due to premature death from illness and years lived with disability. To calculate the DALYs averted in each strategy, the DALYs calculation template from the Health Statistics and Health Information Systems Office (WHO) was used, including the weightings as reported in the Global Burden of Disease 2010 study (Institute for Health Metrics and Evaluation) (33).

The model also includes the probabilities of having a CAD event for the first time, of having another CAD event after the first one, of death at any time and of death because of a CAD event (or due to any cause; see Table 1). Other proposed beneficial effects of lowering TFA intake, such as on insulin sensitivity, obesity, diabetes, cancer, or early growth and development, were not considered in the model because of inconsistent evidence and lack of data (3, 6). The probabilities of having a CAD event were calculated on the basis of the EU hospital discharges (27), because this was the only source of relevant information and data. The lack of CAD incidence data was also highlighted in the 2012 European Cardiovascular Disease Statistics (30), in which hospital discharges were suggested as an alternative source of incidence data. The probabilities of dying at any time and of dying of CAD were extracted from the European Health for All Database (HFA-DB 2010) (27) for the EU.

### Dealing with uncertainty

There is substantial uncertainty with regard to some of the data used in the model. For this reason, we ran the model for various scenarios so as to assess the robustness of the outcome in cases in which data are scarce, in particular with respect to the current EU population's TFA intake. We included 3 scenarios in addition to the base case. In the base case we estimated different initial iTFA intakes per age group and sex (overall average: 0.3 E%):

Scenario 1 assumes an initial overall average iTFA intake of 0.15 E% (50% of our base case estimates, assuming that much improvement has been made since the latest estimates reported in Supplemental Table 1).
Scenario 2 assumes an initial overall average iTFA intake of 0.45 E% (assuming that the situation in countries where no estimates were identified is somewhat worse than in our estimate).
Scenario 3 assumes an initial overall average iTFA intake of 0.7 E% [allowing for even higher initial iTFA intakes as suggested from modeled data of total TFA intake (34) and as presented in **Supplemental Table 4** and after subtracting an estimated 0.5 E% contribution from ruminant TFAs (11)].

Population iTFA intakes in the 3 scenarios diminish in a similar manner as the base case in each of the 4 policy options. A summary of the initial iTFA population intakes for each scenario is provided in **Table 2**.

**TABLE 2:** Overview of different initial iTFA intakes as estimated in the base case and assumed in 3 alternative scenarios^1^

	Initial population iTFA intakes,^2^ E%
Base case	0.3^3^
Scenario 1	0.15
Scenario 2	0.45
Scenario 3	0.7

1 E%, percentage of total energy intake; iTFA, industrially produced *trans* fatty acid.

2 Although the reduction speed differs between the 4 policy options, 0 E% iTFA intake will eventually be achieved in all of the options.

3 Averaged value; initial values in the model in the base case situation differ for age and sex.

In addition, this economic evaluation includes, next to the deterministic analysis, which uses a single value for costs and for effects in the model calculations, a probabilistic sensitivity analysis (PSA). The PSA applies probabilistic distributions to every variable in the model. Each of the distributions used in the PSA were chosen following the current trends and literature recommendations (i.e., γ distribution for variables constrained to be zero or positive or log-normal distribution for variables calculated by using RRs) (31). These probabilistic distributions are based on mean values and CIs, SDs, or ranges of values detailed in the data sources. In this way, the PSA attempts to account for uncertainty in existing evidence [e.g., in the estimates of the risk of CAD linked to different TFA intakes (4)]. A summary of the deterministic values and the distributions applied to them for the PSA analysis are shown in Table 1. Both deterministic and probabilistic analyses were performed for all of the scenarios.

## RESULTS

The resulting health effects and costs linked to each of the 4 policy options from the deterministic base case analysis are presented in **Table 3**. It is important to note that the estimates should not be taken at face value given that the model is a simplification of reality and CAD events and deaths are not “competing” against any other disease. Consequently, the absolute numbers may be an overestimation of CAD events and deaths avoided. This stems from the model’s limitations.

**TABLE 3:** Costs and DALYs associated with 4 policy options to reduce TFA intake in the EU (for the base case)^1^

	Both sexes		Women		Men	
	Costs (× 1 million), €	DALYs (× 1 million)	Costs (× 1 million), €	DALYs (× 1 million)	Costs (× 1 million), €	DALYs (× 1 million)
No action	10,774,890	1077	5,464,667	341	5,310,223	735
Voluntary agreements	10,752,032	1075	5,453,164	341	5,298,867	733
Mandatory labeling	10,870,004	1076	5,513,480	341	5,356,524	734
Legal limit	10,723,635	1073	5,438,734	340	5,284,900	732

1 Values are the result of a deterministic analysis for the full time horizon of the model (85 y) and were calculated by using age and sex specifications. Although costs were similar for men and women, the number of DALYs is nearly double for men. This stems from a difference in ischemic heart disease–related mortality (higher in men), which is reflected in the calculation of DALYs only. DALY, disability-adjusted life-year; EU, European Union; TFA, *trans* fatty acid.

The results shown in Table 3 indicate that implementing a legal limit at the EU level would result in the fewest costs to the public, followed by the voluntary agreements, the reference situation of no-EU-level action, and mandatory labeling. The main reason that the lowest public costs are associated with limiting iTFA contents in foodstuffs is that the reduction in the number of estimated CAD events is greatest due to the lower population intakes of cumulative iTFAs. The reduction in health care costs and in indirect costs linked to informal care and productivity loss outweighs the costs of implementing this policy, more than in any of the other policy options. In contrast, the highest public costs in the mandatory labeling options are due to the fact that the reduction in CAD cases obtained through this policy option is not sufficient to compensate for the costs of the measures implemented. When looking at the health outcomes, the results indicate that introducing an EU-level legal limit on iTFAs in foodstuffs would also result in the smallest number of DALYs. In contrast, taking no action at the EU level (reference situation) would produce the largest number of DALYs, followed by the options of voluntary agreements and mandatory labeling.

To compare the policy options, the difference in costs and DALYs of policy options 2–4 compared with the reference situation (option 1) were calculated and are presented in **Table 4**.These calculations were based on deterministic analyses and were carried out for the base case and the 3 alternative scenarios in which different initial population iTFA intakes were assumed. In addition, the incremental cost-effectiveness ratios (ICERs) for each of the 3 EU-level action policy options are presented in Table 4. The ICER is calculated by dividing the difference in costs between a policy option and the reference situation (no EU-level action) by the respective difference in effects (DALYs); the ICER is then interpreted as the cost for each DALY gained and therefore a lower ICER is preferred.


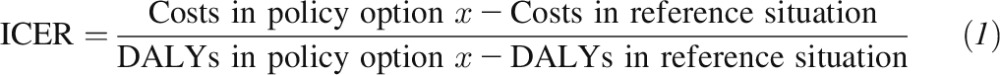


**TABLE 4:** Comparison of the differences in costs and DALYs between the 3 different EU-level action policy options and the reference situation of not acting at the EU level for the base case and the 3 scenarios (deterministic analysis)^1^

	Base case		Scenario 1		Scenario 2		Scenario 3	
	Δ Costs (× million), €	Δ DALYs (× million)	Δ Costs (× million), €	Δ DALYs (× million)	Δ Costs (× million), €	Δ DALYs (× million)	Δ Costs (× million), €	Δ DALYs (× million)
Legal limit vs. no EU action	−51,255	−3.73	−10,686	−1.26	−129,684	−8.59	−279,241	−16.65
Voluntary agreements vs. no EU action	−22,858	−2.19	−2478	−0.75	−70,815	−5.15	−157,017	−10.03
Mandatory labeling vs. no EU action	95,114	−0.98	104,046	−0.42	66,159	−2.90	18,553	−5.62
ICER				
Legal limit	Dominant		Dominant		Dominant		Dominant	
Voluntary agreements	Dominant		Dominant		Dominant		Dominant	
Mandatory labeling	−96,608		−244,913		−22,840		−3301	

1 Values are the result of a deterministic analysis for the full time horizon of the model (85 y). Negative numbers express costs saved and DALYs averted when compared with the reference situation. The absolute value of the ICER (calculated by dividing “Δ Costs” and “Δ DALYs”) represents the cost to the public for each DALY averted for a policy option against the reference of not acting at the EU level. A “dominant” ICER indicates that the policy option in question averts DALYs and saves money. DALY, disability-adjusted life-year; EU, European Union; ICER, incremental cost-effectiveness ratio; Δ Costs, differences in costs; Δ DALYs, difference in DALYs.

A policy option is considered dominant if it can save both costs and DALYs when compared with the reference situation. This is the case for the legal TFA limits and voluntary agreements policy options for the base case and in every scenario considered for our model (Table 4). The legal limit option was found to deliver the highest health benefits and largest cost savings of all EU-level policy options, which remained true in all initial TFA intake scenarios. According to the WHO definition (35), a cost-effective option is that in which the cost-effectiveness ratio is <3 times the Gross Domestic Product (GDP) per capita. Highly cost-effective options are those in which the cost-effectiveness ratio is <1 time the GDP per capita. In the case of the EU, this latter threshold corresponding to the per capita GDP is €23,300. In our evaluation, the mandatory labeling strategy would not be considered cost-effective for the base case or for scenario 1 (lower initial iTFA intakes than in the base case) due to an ICER well above €23,300 and €69,900 (3 times the per capita GDP), whereas this strategy may be cost-effective in cases in which initial iTFA intakes are still relatively high, resulting in an ICER below the threshold (scenarios 2 and 3, assuming higher initial iTFA intakes than in the base case).

**FIGURE 2:**
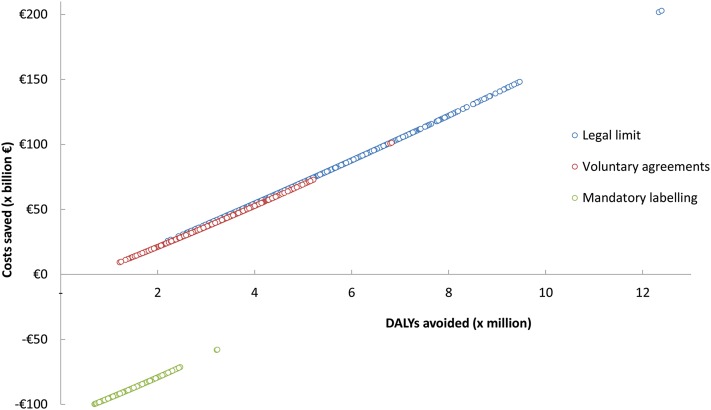
Cost-effectiveness plane. Costs saved against DALYs avoided for each EU-level action policy option against the reference of no EU-level action. The single colored circles represent the outcome of 1 single analysis in the probabilistic sensitivity analysis for each TFA-related EU-level policy option. Each set of colored circles therefore depicts the variation in costs saved against DALYs avoided as a result of the uncertainty in the model input variables. The cost-effectiveness plane is presented for the base case analysis, and values were calculated by using age and sex specifications for the full time horizon of the model (85 y). DALY, disability-adjusted life-year; EU, European Union; TFA, *trans* fatty acid.

Because of the uncertainty associated with the wide distribution of some of the model variable values and data (as described in Table 1), we performed a PSA. Using the model, the analysis was repeated 1000 times, and for each time a random value within the range of values defined in the probability distribution (see Table 1) was used for every input variable. Costs and DALYs were calculated as outcomes for each individual analysis. Importantly, the results obtained with the PSA were similar to those obtained in the deterministic analysis, as shown in **Table 5**. This indicates that variations in the values within the ranges considered do not result in any significant differences in the outcomes of the model. **Table 6** shows the number of times (percentage) in the 1000 outcomes that each policy option was more or less effective than the reference situation. The fact that this sensitivity analysis returns such high (nearly 100%) or low (0%) probabilities reinforces the consistency of our model and the strength of the previous results. **Figure 2** depicts the cost-effectiveness plane of the PSA for the base case scenario.

**TABLE 5:** Comparison of the mean of the differences in costs and DALYs between the 3 different EU-level action policy options and the reference situation of not acting at the EU level for the base case and 3 scenarios (probabilistic sensitivity analysis)^1^

	Base case		Scenario 1		Scenario 2		Scenario 3	
	Δ Costs (× million), €	Δ DALYs (× million)	Δ Costs (× million), €	Δ DALYs (× million)	Δ Costs (× million), €	Δ DALYs (× million)	Δ Costs (× million), €	Δ DALYs (× million)
Legal limit vs. no EU action	−76,478	−5.32	−9,127	−1.13	−144,010	−9.65	−273,864	−16.67
Voluntary agreements vs. no EU action	−35,603	−2.93	−1684	−0.68	−79,067	−5.79	−153,857	−10.04
Mandatory labeling vs. no EU action	89,153	−1.39	104,736	−0.38	59,942	−3.25	20,144	−5.63
ICER				
Legal limit	Dominant		Dominant		Dominant		Dominant	
Voluntary agreements	Dominant		Dominant		Dominant		Dominant	
Mandatory labeling	−64,363		−274,163		−18,433		−3580	

1 Values are the means of 1000 outcomes in a probabilistic sensitivity analysis for the full time horizon of the model (85 y). Negative numbers express savings when compared with the reference situation. The absolute value of the ICER (calculated by dividing “Δ Costs” and “Δ DALYs”) represents the cost to the public for each DALY averted for a policy option against the reference of not acting at the EU level. A “dominant” ICER indicates that the policy option in question averts DALYs and saves money. DALY, disability-adjusted life-year; EU, European Union; ICER, incremental cost-effectiveness ratio; Δ Costs, differences in costs; Δ DALYs, difference in DALYs.

**TABLE 6:** Probabilities of each EU-level action policy option to save costs and DALYs when compared with the reference situation in the base case and 3 scenarios^1^

	Base case, %		Scenario 1, %		Scenario 2, %		Scenario 3, %	
No EU action compared with	Probability of saving costs	Probability of saving DALYs	Probability of saving costs	Probability of saving DALYs	Probability of saving costs	Probability of saving DALYs	Probability of saving costs	Probability of saving DALYs
Voluntary agreements	100	100	90.6	100	100	100	100	100
Mandatory labeling	0	100	0	100	0	100	0	100
Legal limit	100	100	100	100	100	100	100	100

1 Values refer to the probabilistic sensitivity analysis for the full time horizon of the model (85 y). DALY, disability-adjusted life-year; EU, European Union.

The estimates confirmed that legal limit and voluntary agreements are the policy options in which more costs are saved and more DALYs are avoided than in the reference situation. These results are robust because the probability of saving costs and DALYs in the PSA is 100%. In the base case scenario, mandatory labeling does not appear to save costs but would also avoid DALYs in 100% of the trials.

Overall, the estimates in Table 5 indicate that imposing legal limits on the iTFA content in foods could save ∼ €76 billion and avoid 5.32 million DALYs over an 85-y period compared with the current situation, with a 100% of probability of being cost-effective. The similarity between the direction of the results obtained in the PSA and the deterministic analyses highlights the robustness of the model, although values in the PSA are higher than in the deterministic analysis. Both show that the 3 alternative EU-level action policy options are more effective than the reference situation of not acting at the EU level. As in the deterministic analysis, the PSA confirms that legal limits and voluntary agreements strategies are dominant (i.e., they provide health benefits and save costs), whereas mandatory labeling is highly cost-effective (in terms of GDP per capita threshold) only in scenarios 2 and 3, assuming higher (0.45 and 0.7 E%) than base case estimated initial iTFA intakes.

## DISCUSSION

The results of the model indicate that both introducing an EU-level legal limit and making voluntary agreements would save money and provide additional health benefits (avoiding DALYs) compared with not taking action at the EU level. Note also that although our analysis is focused on the EU market, it is likely that any action at the EU level would also affect the presence of iTFAs in foods and iTFA intake in other non-EU countries.

Despite a variety of uncertainties associated with some of the data included in the model, the PSA suggests that these results are robust because both policy options are dominant (saving costs and DALYs) in 100% of the trials in the PSA and the CIs are quite narrow. The same occurred for every scenario tested, except for the voluntary agreements in scenario 1, which assumed the lowest initial population iTFA intake of only 0.15 E% (90.6% probability). Although important, the cost-effectiveness of a particular policy option is not the only variable to be considered by policy makers to implement new policies. For example, our model focused on public expenditure and did not contemplate any potential costs incurred by the industry or other players when limiting iTFA content in foods. This is common practice in public health economic evaluations (25). In addition, neither EU member states nor EU stakeholders have pinpointed costs related to removing partially hydrogenated oils (PHOs) from foods (22). This is probably linked to the gradual progress in the innovation of PHO alternatives and the fact that the EU food industry has, over time, already removed PHOs from food products to a large extent (32).

Although our model applied a lifetime horizon (85 y) for calculating health effects and costs, others used shorter time horizons (15–18). The time horizon needs to be sufficiently long to reflect all important differences in costs or outcomes between the policy options under comparison (25). We decided that a lifetime time horizon was appropriate because the varying iTFA intakes linked to the 4 policy options led to differences in survival and benefits that persist throughout a person's life.

Introducing mandatory TFA labeling at the EU level, regardless of the scenario analyzed, also has the potential to avert DALYs but not costs. In general, the differences in costs between the different policy options are driven by the costs related to the incidence of CAD. However, in cases in which the difference in CAD incidence between an alternative option and the reference situation is low, such as the case for mandatory TFA labeling, the leading costs then stem from the costs of the measures (school-based interventions, worksite interventions, mass media campaigns, physician counseling, and food inspection programs).

With regard to the differences in DALYs, these are mostly due to the number of CAD events and related premature deaths, as well as the number of years living with disability and the number of years lost because of premature death. Small resulting differences in CAD are particularly likely in scenario 1, in which the lowest initial iTFA intakes are assumed.

In this study, we assumed a rather rapid removal of iTFAs from the EU food supply over 10 y, including in the reference situation in which no EU-level action is taken (we assumed efforts at the national level instead). This rather optimistic assumption for the reference situation means that we used a conservative approach in our conclusions on the EU-level action policy options. Health benefits would be even larger and cost savings more likely should iTFA intakes decrease more slowly than what is assumed in the reference situation in which no EU-level action is taken.

As discussed previously, some of the model variables or input data used here are rather uncertain and several assumptions had to be made. There are 3 major sources of potential errors: the estimated current TFA intake, the wide variability observed for many variables between countries, and the lack of data in some instances, such as the lack of data on the number of CAD events per year (CAD-related hospital discharges were used instead). To address the concerns related to the accuracy of estimated iTFA intake values entered in the model, we used different scenarios with different values for initial iTFA intake. In all of the scenarios and for all policy options it was assumed that, with time, iTFA intakes will eventually decrease to 0 E%. This seems reasonable given the current estimates of iTFA intakes of ∼0.01 E% for Denmark, where a legal limit on iTFA contents of foods has been in place for 10 y (see Supplemental Tables 1 and 2 and reference 22). The wide variability in the data between European countries and the absence of reliable data on the incidence of CAD are addressed with the PSA. As discussed above, the robustness of the results obtained with the PSA indicates that, despite the uncertainties associated with the input data, the outcome is still valid and reliable. Again, however, we note that the absolute numbers estimated by the model are likely to overestimate the number of CAD events.

The results presented here should be interpreted as a comparison between different policy options rather than considering absolute costs, DALYs, or deaths per option. Previous work has shown the benefits of TFA intake reduction (18). Recently, the Food and Drug Administration also released a “final determination” that PHOs, the primary dietary source of iTFAs, are no longer considered to be “generally recognized as safe” products (36). This was accompanied by a memorandum that estimated costs and potential health effects of limiting iTFA content in foods in the United States (17). The authors indicated that “monetizing the lives saved, along with the value of the nonfatal illnesses and medical expenses prevented, yields an estimated benefit of $14.7 billion/y, starting 3 y after the elimination of partially hydrogenated oils from the food supply.” Although the model used and the real costs and DALYs calculated in that memorandum differ considerably from those presented here, the conclusions are similar, namely that removing iTFAs from the US food supply would save costs and DALYs.

In conclusion, the results of this study suggest additional health benefits and reductions in public spending when taking EU-level action toward reducing population iTFA intakes. Although now introducing a mandatory TFA labeling scheme may not be a cost-effective solution in the EU, both a legal limit on the iTFA content in foods and voluntary agreements toward removing PHOs from foods produce large-enough reductions in CAD morbidity and mortality that the related reductions in costs outweigh the costs linked to the implementation of these strategies. Finally, introducing a legal limit at the EU level would produce the greatest health benefits of all of the options included in this study.

## Footnotes

4 Abbreviations used: CAD, coronary artery disease; DALY, disability-adjusted life-year; E%, percentage of total energy; EU, European Union; GDP, Gross Domestic Product; ICER, incremental cost-effectiveness ratio; iTFA, industrially produced *trans* fatty acid; PHO, partially hydrogenated oil; PSA, probabilistic sensitivity analysis; TFA, *trans* fatty acid.
